# Editorial: Aging with neurodevelopmental disorders (NDD)

**DOI:** 10.3389/fnint.2023.1167014

**Published:** 2023-03-02

**Authors:** Zheng Wang, Matthew W. Mosconi

**Affiliations:** ^1^Department of Applied Physiology and Kinesiology, University of Florida, Gainesville, FL, United States; ^2^Clinical Child Psychology Program, University of Kansas, Lawrence, KS, United States; ^3^Life Span Institute, University of Kansas, Lawrence, KS, United States; ^4^Kansas Center for Autism Research and Training, University of Kansas, Lawrence, KS, United States

**Keywords:** neurodevelopmental disorders (NDDs), aging, *FMR1* premutation carrier, Down Syndrome, Cerebral Palsy

## Introduction

Neurodevelopmental Disorders (NDDs) represent a broad class of conditions characterized by difficulties in cognitive, communicative, behavioral or adaptive skills that manifest early in development and persist across the lifespan. Considerable research attention has been dedicated to understanding the causes and early emerging developmental profiles of different NDDs, but there is a striking paucity of research focused on disease progression through adulthood.

These large gaps are particularly salient in the context of findings showing strong associations between NDDs and risks for both health issues (Sundahl et al., 2016; Whitney et al., 2020) during adulthood and neurodegenerative disease during aging (Holland et al., 2000; Starkstein et al., 2015; McCarron et al., 2017). As the baby boomer generation reaches middle and older adult ages and the number of adults living with an NDD continues to increase, the need to address gaps in identifying risks for health issues and monitoring aging-associated comorbidities in adults with NDDs becomes increasingly urgent. NDD communities, researchers, clinicians, stakeholders, and policymakers each have raised concerns about the consequences of aging in NDDs and identified “meeting the needs of people with NDDs as they progress into and through adulthood” as a critical healthcare and research priority (Roestorf et al., 2019; Interagency Autism Coordinating Committee, 2020). This Research Topic aims to address these gaps and help highlight the critical need for research on aging processes in NDDs. Four diverse papers are included covering separate issues in adults with NDDs, including carriers of premutation alleles of the Fragile X gene (*FMR1*), people with Down Syndrome, and individuals with Cerebral Palsy. Our goal is to heighten awareness of the unique and diverse needs of adults living with an NDD.

To assess neuropsychiatric issues in adult female premutation carriers of the Fragile X gene, *FMR1*, Schmitt et al. conducted a dense phenotyping study including self-report, eye tracking, quantitative experimental, and resting state electroencephalogram (rs-EEG) tests. *FMR1* premutations are associated with multiple subclinical and clinical issues during adulthood, including Fragile X-associated Tremor and Ataxia Syndrome (FXTAS), Fragile X-associated Primary Ovarian Insufficiency (FXPOI), and Fragile X-associated Neuropsychiatric Disorder (FXAND) (Hagerman et al., 2018; Salcedo-Arellano et al., 2020). While premutation alleles appear to be less penetrant for clinical degeneration among females relative to males, they still appear to confer significant risk for medical and neuropsychiatric impairments. Understanding pathways associated with risk for neuropsychiatric issues among female premutation carriers will be critical for determining distinct genetic and neurophysiological signatures predicting different clinical outcomes. The authors report on a well-characterized cohort and, using a data-driven cluster analysis approach, identified three distinct phenotypic profiles, or “clusters”. Cluster 1 (27%) manifested elevated psychiatric symptoms, including anxiety and depression, high CGG repeat counts, and typical rs-EEG features. Cluster 2 (32%) demonstrated elevated executive dysfunction and an atypical EEG profile of reduced Theta and increased Gamma1 and Gamma2 bands relative to controls. Cluster 3 (41%) represented a relatively “unaffected” group with minimal abnormalities identified across all testing domains. These findings offer important new information on genetic and neurophysiological substrates that may contribute to neuropsychiatric issues in adulthood among female premutation carriers.

Building on these findings and studying an overlapping cohort of female *FMR1* premutation carriers, Norris et al. used a quantitative EEG approach to examine neurophysiological differences associated with auditory processing. While multiple studies have reported sensory abnormalities in premutation carriers during childhood, few studies have investigated sensory processing in adult premutation carriers. Results from this study showed the cohorts established by Schmitt et al. also demonstrated distinct auditory neural signatures during processing of a “chirp” stimulus, including decreased response to stimulus onset and a low sensory registration score among the “psychiatric” Cluster 1, decreased Gamma phase locking to the chirp stimulus and high sensory sensitivity in the “executive dysfunction” Cluster 2, and relatively intact EEG profiles in the “unaffected” Cluster 3. Combined, these studies provide new information on key neural signatures that may be useful for parsing clinical heterogeneity in adult female premutation carriers.

To understand the unique needs of adults with Down Syndrome (DS), Peven et al. examined the relationship between physical activity and memory function. Associations between DS and Alzheimer's disease (AD) are now well-documented, so factors that can mitigate declines in cognitive and memory abilities are critical for slowing disease progression. Physical activity may be a feasible target for this purpose because it has been shown to be effective in improving memory function and lowering risk for dementia in neurotypical adults (Erickson et al., 2011; Hoffmann et al., 2021). To assess associations between physical activity and memory function, the authors studied 81 adults (mean age: 38 yrs; SD: 8 yrs) with DS who participated in a 7-day ancillary Lifestyle program. Greater engagement in moderate physical activity and increased hippocampal volume each were associated with better episodic memory in individuals with DS, suggesting that lifestyle changes may serve to mitigate cognitive degeneration during adulthood. Prospective follow-up studies will be important for determining whether physical activity leads to lower risk or later onset of AD in adults with DS.

Whitney et al. provided a prospective review assessing the extent to which well-established clinical measures predicting health outcomes for the general population may not apply to adults with Cerebral Palsy (CP). CP is associated with multiple disease-specific anatomical and physiological changes that confound clinical guidelines during translation. For example, lower body masses common to adults with CP lead to lower body mass index (BMI) scores despite high rates of obesity in this population. Bone mineral density (BMD) and glomerular filtration rate (GFR) are additional metrics useful for risk assessments of bone fracture and kidney dysfunction that may require separate consideration when interpreted with adults with CP. This prospective review emphasizes the need for differential interpretation of health metrics for adults with CP and other forms of NDDs. The authors also make the case that applying clinical standards to NDD populations requires integrating the unique characteristics of the individuals being evaluated.

## Conclusion and future directions

Articles in this Research Topic each highlight the urgent need for greater research attention on the unique needs and challenges of individuals with NDDs as they enter adulthood. As seen in childhood, outcomes associated with different NDDs show high levels of variability across individuals. Impairments caused by NDDs also appear to change significantly across the lifespan, indicating that new support strategies will be needed, and approaches for clinically monitoring individuals with NDDs as they age will need refinement. This refinement process should be based on rigorous and comprehensive studies of the natural history of different NDDs across the full lifespan, and the incorporation of measurements of the environmental and neurobiological factors that may affect individuals beyond childhood. We thank all authors for their significant contributions and reviewers for their constructive feedback. We hope that this Research Topic stimulates fruitful discussion and vigorous research to promote better understanding of and supports around aging-related changes in individuals with NDDs.

## Author contributions

ZW drafted the editorial. MM revised the editorial. All authors have made a direct contribution to the work and approved it for publication.
